# Evaluation of the Immune Response Afforded by Combined Immunization with Orf Virus DNA and Subunit Vaccine in Mice

**DOI:** 10.3390/vaccines10091499

**Published:** 2022-09-08

**Authors:** Yan Wang, Kui Zhao, Deguang Song, Le Du, Xinyue Wang, Feng Gao, Huijun Lu, Jiyu Guan

**Affiliations:** 1 Key Laboratory of Zoonosis, Ministry of Education, College of Veterinary Medicine, Jilin University, Changchun 130062, China; 2 Key Laboratory of Zoonosis, Ministry of Education, Institute of Zoonosis, Jilin University, Changchun 130062, China

**Keywords:** Orf virus, DNA prime-protein boost immunization, immune responses

## Abstract

Contagious ecthyma (Orf) is a highly contagious disease caused by Orf virus (ORFV) infection. Orf is prevalent all over the world and, not only affects the healthy development of sheep husbandry, but also threatens human health. However, there are no safe and effective vaccines or drugs for the prevention and treatment of Orf at present. In this study, we constructed a DNA plasmid expressing ORFV *B2L* and *F1L* genes as a DNA vaccine candidate, with purified *B2L* full-length protein and *F1L* truncated protein as subunit vaccine candidates. BALB/c mice were immunized with the DNA vaccine, subunit vaccine, as well as DNA prime-protein boost strategies. The results showed that compared with the DNA vaccine and subunit vaccine alone, the DNA prime-protein boost immunization group had a higher level of specific antibodies, stronger lymphocyte proliferation, and higher expression of cytokines such as IL-2, IL-4, IL-6, IFN-γ, and TNF-α, which are considered to cause a Th1/Th2 mixed cytokine response. Our results demonstrated that the DNA prime-protein boost immunization strategy induced stronger humoral and cellular immune responses, which have potential advantages in preventing ORFV infection.

## 1. Introduction

Contagious ecthyma (Orf) is a highly contagious disease caused by Orf virus (ORFV) infection, and it is also a zoonotic disease that occurs all over the world [1,2]. Animals infected by ORFV are mainly sheep and goats, with cats, camels, musk ox, and so on occasionally infected, the host range of infection is expanding [3]. In clinically infected animals, papules, pustules, and crusted lesions appear on the lips, gums, and nose [4], making it difficult for the sick animals to swallow food, resulting in death and economic losses. Although the mortality rate of sick adult sheep is relatively low, the incidence rate in lambs due to secondary infection can reach up to 93.7%, and the mortality rate can reach 15% [5,6]. ORFV can also infect people who have had extensive contact with affected animals, such as farmers, animal managers, and veterinarians [7,8,9]. In a British report, the survey found that 30% of shepherds had been infected with ORFV [10]. Therefore, ORFV not only affects the development of sheep husbandry, but also threatens human and animal health.

At present, there are no commercially available therapeutic agents for Orf. Treatment with topical antibiotic ointment in Orf virus infected animals is considered effective for the prevention of the secondary infection by bacteria, alleviating the clinical symptoms caused by Orf disease. Nevertheless, this can lead to drug resistance and drug residues in animals, which affects the treatment of other diseases [11]. Therefore, vaccines are the best alternative to antibiotic drugs. Until now, there has been no vaccine for this disease, and it would be of great value to develop a safe and effective vaccine. Whether vaccines can exert good immune efficacy and provide effective protection depend on appropriate antigen selection, which is the key for vaccine development. ORFV belongs to the Poxviridae family, the parapoxvirus genus, a double-stranded DNA virus that encodes about 134 genes. ORFs 009-111 is the central core region of the virus and is highly conserved; it is involved in viral, maturation, and virion structure formation and morphology, which play an important role in the replication and maturation of the virus and the formation and morphogenesis of virus particles [1]. ORFV *B2L* gene encoded 42 kDa protein is an important immunogenic protein that induces a strong antibody response [12]. ORFV *F1L* gene is involved in the transcriptional coding in the late stage of viral infection and can be expressed as an immune protein on the envelope of the mature virion. Studies have found that both the full-length *F1L* protein [13] and the truncated *F1L* protein (with the transmembrane region removed) [14] have good immunogenicity and can stimulate antibody production, making it a candidate antigen for a subunit vaccine. A previous study found that recombinant DNA vaccines with the chimeric expression of the ORFV *B2L* and *F1L* genes induced higher levels of antibodies in mice than single-gene recombinant DNA vaccines, indicating that the combination of two immunogenic genes as antigens can produce a satisfactory effect [15]. Additionally, similar results were found in some reports about other kinds of viral vaccines. For example, when mice were immunized with recombinant adenovirus taking two immunogenic genes of porcine reproductive and respiratory syndrome virus, the combined immunization of two-gene adenovirus vaccine produced higher levels of antibodies than of the single-gene adenovirus vaccine [16]. In a study on the immune effect of the subunit vaccine of bovine viral diarrhea, the combined immunization of mice with two virus-encoding immunogenic proteins was better than that of a single protein [17]. Therefore, in the context of Orf vaccine development, there are potential advantages in integrating *B2L* and *F1L* genes simultaneously in vaccines.

Compared with traditional vaccines, DNA vaccines can deliver a variety of important antigens through the same expression vector, which can trigger humoral and cellular immunity in the body. This has the advantages of simple operation, good stability, no infection, low production cost, short development, and production cycle [18]. Subunit vaccines are prepared by extracting or synthesizing the immunogenic protein of the pathogens, which has the advantages of safety, low production cost, and good immune effect [19]. However, the protective effect of single-type vaccine immunization still needs to be improved. Several studies have shown that heterologous priming-boosting can enhance the immune protection of animals and that this is one of the best strategies to improve the immune efficiency of DNA or subunit vaccines. For example, compared with the single use of DNA vaccine or subunit vaccine, the immunization method of heterologous immunization enhanced the protective effects of chickens against Newcastle disease virus infection [20], of pigs against highly infectious foot-and-mouth disease virus infection [21], and of mice against Middle East respiratory syndrome coronavirus (MERS-COV) infection [22]. Mixing adjuvant with antigen for animal immunization can prolong the period of immune interaction between the antigen and the body, stimulate the body to continuously produce antibodies, and finally make the body obtain higher levels of antibodies [23]. Freund’s adjuvant is the most commonly used water-in-oil adjuvant in animal experiments, which has a strong capacity to stimulate the immune system, to produce a strong immune response together with the antigen [24].

In this study, we used ORFV (SY17 strain) encoded *B2L* and *F1L* proteins as immunogens for the preparation of DNA and subunit vaccines. Then we evaluated the effects of DNA/protein, Protein/Protein, and DNA/DNA immunization strategies on humoral and cellular immunity in mice.

## 2. Materials and Methods

### 2.1. Viruses, Plasmids, and Cells

ORFV, pcDNA3.1 (+), pEGFP-C3, pET-30a, and pET-32a vectors were all preserved in our lab. Primary ovine fetal turbinate (OFTu) cells were maintained in Dulbecco’s modified eagle medium (DMEM) (Meilunbio, Dalian, China) supplemented with 10% fetal bovine serum (FBS) (Biological Industries, Beit HaEmek, Israel), at 37 °C with 5% CO_2_.

### 2.2. Animals

Six-week-old female BALB/c mice weighing 18–20 g were purchased from Changsheng Biotechnology Co., Ltd. in Liaoning, China. All the mice used in the study were chosen randomly. The animal experiments were approved by the Animal Ethics Committee of the College of Veterinary Medicine of Jilin University.

### 2.3. Construction of Vaccine Vector

According to the GeneBank accession number MG712417.1 published by NCBI, Primer premier 5.0 software was applied to design specific PCR primers, and primers were provided by the Kumei biological company (Table 1). PrimeSTAR Max (Takara Bio, Kusatsu, Japan) was used to amplify the *B2L* and *F1L* genes and connect them through the DNA sequence of P2A; in order to verify their expression, HA, Flag, and mCherry sequences were introduced into the beginning of these genes. They were cloned into the linearized pcDNA3.1 (+) and pEGFP-C3 vectors using homologous recombinase (TransGen Biotech, Beijing, China) and named plasmids pcDNA3.1-*B2L*-P2A-*F1L*, pcDNA3.1-HA-*B2L*-P2A-Flag-*F1L*, and pEGFP-C3-*B2L*-P2A-mCherry-*F1L*.

The full length of *B2L* and the extracellular segment of the *F1L* (coding 1-260 aa of *F1L*) gene without the transmembrane region were cloned into pET-32a and pET-30a prokaryotic expression plasmids, named pET-32a-*B2L* and pET30a-c*F1L*, respectively. These plasmids were identified by sequencing and enzyme digestion assay. All of the plasmids were extracted using an endotoxin removal plasmid kit (TianGen Biotech, Beijing, China). DNA concentration was determined with a nucleic acid concentration analyzer (NanoPhotometer-N50, Munich, Germany).

### 2.4. Immunofluorescence Assay

In order to verify the protein expression of the recombinant eukaryotic expression plasmid. OFTu cells were cultured in 12-well plates with cell slides until the confluence reached 70%. The pEGFP-C3 vector control and pEGFP-C3-*B2L*-P2A-mCherry-*F1L* plasmid were transfected into OFTu cells, incubated in a CO_2_ incubator at 37 °C for 48 h, and phosphate-buffered saline (PBS) was used to wash cells three times; they were fixed with 4% paraformaldehyde at room temperature (RT) for 15 min; then, the cells were washed with PBS three times; the cell slides were removed, using 4′, 6-diamidino-2-phenylindole (DAPI) for nuclei staining at RT for 10 min; finally, the results were examined by confocal microscopy.

### 2.5. Transfection of OFTu Cells

To verify the cleavage of the self-cleaving peptide P2A and the protein expression of the eukaryotic expression plasmid. The OFTu cells were passaged to a 6-well plate. After 18–24 h, the confluence reached about 70%, and the cells were transfected with pcDNA3.1 (+) vector and pcDNA3.1-HA-*B2L*-P2A-Flag-*F1L* plasmids were incubated in a CO_2_ incubator at 37 °C for 48 h; cells were lysed with a mixture of RIPA (Beyotime, Shanghai, China) lysate and protease inhibitor (PMSF) and lysed on ice for 30 min; centrifuged at 12,000 rpm for 10 min at 4 °C; a part of the supernatant was collected, and the protein concentration was determined with a BCA protein assay kit (Beyotime, Shanghai, China); SDS-PAGE loading buffer was added to another part of the supernatant, boiled for 8 min, and stored at −20 °C for later use.

### 2.6. Preparation of Recombinant Proteins B2L and cF1L

Recombinant protective antigens were expressed and purified in *E. coli* cells, and the synthetic pET-32a-*B2L* and pET-30a-c*F1L* (1-260 aa) were transformed into BL21(DE3)pLysS chemically competent cell. The monoclonal colony was selected; inoculate into 4 mL of liquid medium containing LB, at 37 °C, 180 rpm; and cultivated to an OD_600_ value of 0.4–0.6. Shaken bacterial solution was added to the LB liquid medium with the same resistance in the ratio of 1:20, and expression was induced with 0.2 mM IPTG for 10 h; then, purified with nickel chelate affinity chromatography. pET-32a-*B2L* is an inclusion body protein. The purified protein was put into a dialysis bag, and then renatured in a renaturing solution containing 6, 4, 2, and 0 M urea [25]. pET-30a-c*F1L* is a soluble protein and is normally purified. The purity of the two purified proteins was detected by SDS-PAGE; the protein concentration was detected using a BCA protein assay kit (Beyotime, Shanghai, China); immunogenicity was detected by Western blotting.

### 2.7. Western Blotting

The purified *B2L*, c*F1L* proteins, pcDNA3.1 (+) plasmid transfected OFTu cell lysates, pcDNA3.1-HA-*B2L*-P2A-Flag-*F1L* transfected OFTu cell lysates, and untransfected OFTu cell lysates were collected, then all of them were added to SDS-PAGE loading buffer (Beyotime, Shanghai, China), boiled in water for 8 min; 10% protein gel was used to separate proteins. Proteins were transferred to nitrocellulose membrane (Merck, Darmstadt, Germany); 5% nonfat dry milk was blocked at 37 °C for 1 h; the membranes with *B2L* and c*F1L* proteins were incubated with mouse polyclonal antibodies in the ratio of 1:1000, and all transfected and untransfected OFTu cell lysates were incubated with anti-HA and anti-Flag tag mouse monoclonal antibodies in a ratio of 1:1000. The primary antibody was incubated at 4 °C overnight, washed three times with PBS-T buffer, then, following detection with 1:10,000 of horse radish peroxidase (HRP)-conjugated goat anti-mouse IgG antibody, incubated at 37 °C for 1 h (Proteintech, Chicago, USA). It was washed three times with PBS-T buffer; ECL supersensitive luminescence developer was added for development, and the results were recorded by taking pictures with a developer (Tanon, Shanghai, China).

### 2.8. Animal Immunization

A total of 50 SPF female BALB/c mice (6-week-old age) were randomly divided into 4 groups (*n* = 10) with 10 animals in each group: namely, a PBS group, DNA/DNA group, Protein/Protein group, and DNA/Protein group. Mice were immunized on day 0 and day 21. For the PBS control group, immunization was performed twice with 100 μL PBS in each treatment; for the DNA/DNA group, twice with 100 μg/100 μL pcDNA3.1-*B2L*-P2A-*F1L* DNA vaccine at indicated time points; for the Protein/Protein group, twice with 20 μg/50 μL *B2L* and 20 μg/50 μL c*F1L* proteins at indicated time points; for DNA/Protein group, the first time with 100 μg/100 μL pcDNA3.1-*B2L*-P2A-*F1L* DNA vaccine, the second time with 20 μg/50 μL *B2L* and 20 μg/50 μL c*F1L* proteins at indicated time points. The immunization method of the DNA vaccine was intramuscular injection, and the immunization method of subunit vaccine was subcutaneous injection. The volume ratio of adjuvant to antigen was 1:1. Complete Freund’s adjuvant was used for the first immunization, and the amount of complete Freund’s adjuvant was 100 μL when combined with the DNA vaccine. Incomplete Freund’s adjuvant was used for booster immunization. The amount of incomplete Freund’s adjuvant was 100 μL when combined with the subunit vaccine. The experimental period was 35 d, the details of mouse vaccination are shown in Table 2.

### 2.9. Preparation of Polyclonal Antibodies

The purified recombinant protein was mixed with Freund’s complete adjuvant in an equal volume of 1:1, shaken at 4 °C for emulsification overnight, and then dropped into the water surface after emulsification; the emulsification was successful if it did not spread after forming a mass. The first immunization of BABL/c mice by subcutaneous injection was carried out at multiple points on the back, and the amount of immunized protein in each mouse was 150 μg; on days 7, 14, and 28 after the first immunization, the purified recombinant protein was emulsified with an equal volume of incomplete Freund’s adjuvant and subcutaneously injected again at multiple points for immunization, and the immunization dose was 100 μg per mouse. On day 10 after the fourth immunization, the blood of the immunized mice was collected from the retro-orbital vein, and the serum was collected and stored at −80 °C for future use.

### 2.10. Specific Antibody Detection

The sera of mice were collected on day 0, 7, 14, 21, 28, and 35 after the initial vaccination, and the levels of immunogen-specific antibodies in the sera of immunized mice were measured by indirect ELISA. The optimal concentration of protein coating was determined by the checkerboard method. The purified *B2L* and c*F1L* proteins were diluted with pH 9.6 carbonate buffer to 3 μg/mL, and then added to a 96-well enzyme in an amount of 100 μL/well. The target plate was coated overnight at 4 °C, the liquid was discarded, 5% skimmed milk powder was added, at 200 μL/well, and 37 °C was blocked for 1 h. The liquid was discarded, and the plate was washed three times with PBS-T buffer, 5 min each time, and patted dry. The serum to be tested was diluted 1:100, each sample was replicated three times, 100 μL/well and a blank control well, and positive control (polyclonal antibody serum) was set, at 37 °C for 1 h. The liquid was discarded, the plate was washed three times with PBS-T buffer, for 5 min each time, and patted dry. After the horse radish peroxidase (HRP)-conjugated goat anti-mouse IgG antibody was diluted with PBS-T buffer, each well was supplemented with 100 μL diluted antibody, and incubated at 37 °C for 1 h. The liquid was discarded, the plate was washed three times with PBS-T buffer, 5 min each time, and patted dry. Color developing solution was added to each well; stop solution was added after 5–15 min of color development in the dark, and the OD value was read at 450 nm.

### 2.11. Specific Antibody IgG Subclass Detection

The levels of IgG1 and IgG2a in mouse serum were detected using a mouse IgG1 and IgG2a ELISA Kit (Jiang Lai, Shanghai, China) and according to the reagent instructions.

### 2.12. Spleen Lymphocyte Proliferation Assay

To study the proliferation activity of immune cells, on day 14, after the second immunization, five mice in the experimental groups and five mice in the control group were randomly selected, and their spleen cells were isolated and added to RPMI-1640 (Meilun Bio, Dalian, China) complete culture medium. The cells were stained with trypan blue and counted under a microscope, and the concentration of cells was adjusted to 1 × 10^7^/mL. The prepared mouse spleen lymphocytes were added to a 96-well cell culture plate, and 100 μL of cell suspension was added to each well. Three replicates per mouse were set up, 5 μg/mL of purified *B2L* and c*F1L* protein were added to each group, the complete medium was used as a blank control group, and a final concentration of 5 μg/mL was stimulated with ConA as a positive control and cultured in 5% CO_2_ at 37 °C. After 68 h, 10 μL MTT (5 mg/mL) was added to each well and cultured for 4 h, the culture plate was removed, 100 μL DMSO was added to each well, and placed in the dark at room temperature for 15 min to measure the absorbance at OD_450nm_. The mean value was calculated, and the side score index was calculated.

### 2.13. Cytokine Detection

On day 14, after boosting immunization, mouse spleen lymphocytes were stimulated with 5 μg/mL purified *B2L* and c*F1L* proteins, and the culture supernatants were collected. Mouse IL-2, IL-4, IL-6, IFN-γ, and TNF-α ELISA Kits (Biolegend, San Diego, CA, USA) were used to detect the content of cytokines secreted into the supernatant of stimulated splenocytes, which were detected according to the reagent instructions.

### 2.14. H&E Staining

On day 14, after boosting immunization, the mice were euthanized; and the heart, liver, lung, and kidney were collected and placed in 4% paraformaldehyde for fixation at room temperature for 48 h. Then they were dehydrated, embedded in paraffin, stained with hematoxylin and eosin, mounted, and observed under a light microscope (Leica DM4000B, Wetzlar, Germany).

### 2.15. Statistical Analysis

All experimental data were analyzed for differences between groups using *t*-test or one-way ANOVA in GraphPad Prism 6.0 software (San Diego, CA, USA), the data are expressed as mean ± standard error, * *p* < 0.05, ** *p* < 0.01, and *** *p* < 0.001 were considered statistically significant.

## 3. Results

### 3.1. Construction of DNA Vaccine Expressing B2L/F1L Fusion Protein

**Figure 1:**
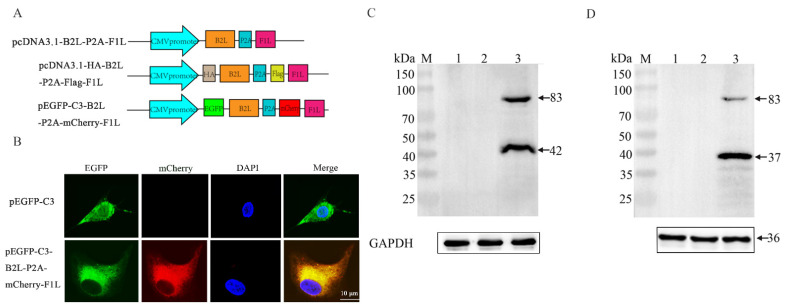
Construction of DNA vaccine expressing *B2L*/*F1L* fusion protein. (**A**) Schematic diagram of pcDNA3.1-*B2L*-P2A-*F1L* plasmid, pcDNA3.1-HA-*B2L*-P2A-Flag-*F1L* plasmid, and pEGFP-C3-*B2L*-P2A-mCherry-*F1L* plasmid. (**B**) Indirect immunofluorescence detection of fusion recombinant plasmids transfected into OFTU cells. The magnification is 1000×, Scale bars: 10 μm. (**C**) Western blotting of OFTu cells transfected with pcDNA3.1-HA-*B2L*-P2A-Flag-*F1L* plasmid. Membrane incubated with anti-HA tag mouse monoclonal antibodies. Lane 1: OFTu cell lysates, lane 2, and lane 3: the transfected pcDNA3.1 (+) plasmid and pcDNA3.1-HA-*B2L*-P2A-Flag-*F1L* plasmid cell lysates. (**D**) Western blotting of OFTu cells transfected with pcDNA3.1-HA-*B2L*-P2A-Flag-*F1L* plasmid. Membrane incubated with anti-HA tag mouse monoclonal antibodies. Lane 2 and lane 3: the same sample was used as in (**C**). M: protein molecular weight marker.

For constructing a DNA vaccine expressing ORFV *B2L*/*F1L* fusion protein, pcDNA3.1-*B2L*-P2A-*F1L* was designed and constructed (Figure 1A). To confirm the expression of *B2L*/*F1L* in the cells, pEGFP-C3-*B2L*-P2A-mCherry-*F1L* plasmid was designed and constructed, as shown in Figure 1A. pEGFP-C3-*B2L*-P2A-mCherry-*F1L* and control plasmid pEGFP-C3 were transfected into OFTu cells for 24 h, which was followed by observation of the fluorescence signal in the cells. Compared with the cells in the control group, the cells transfected with pEGFP-C3-*B2L*-P2A-mCherry-*F1L* displayed both green and red fluorescence signals (Figure 1B).

To verify whether *B2L*/*F1L* fusion protein could be expressed separately under the cleavage of P2A peptide, pcDNA3.1-HA-*B2L*-P2A-Flag-*F1L* was designed and constructed (Figure 1A). pcDNA3.1-HA-*B2L*-P2A-Flag-*F1L* was transfected into OFTu cells for 48 h, which was followed by cell collection. The cell lysates were verified by Western blotting, as shown in Figure 1C,D. The HA-tagged *B2L* protein (42 kDa) could be detected when the anti-HA antibody was incubated with the sample (Figure 1C); The Flag-tagged *F1L* protein (37 kDa) could be detected when anti-Flag antibody was incubated with the sample (Figure 1D). This data demonstrates that, due to the presence of the 2A peptide, the fusion protein could be auto-cleaved into two separate proteins. Notably, the 83 kDa protein was an unsuccessfully cleaved fusion protein, suggesting that the cleavage efficiency was not 100% (Figure 1D). There were no specific bands in the empty and untransfected OFTu cells. In summary, the above data indicate that the *B2L* and *F1L* proteins could be independently expressed in OFTu cells.

### 3.2. Expression and Purification of B2L and cF1L Protein

**Figure 2:**
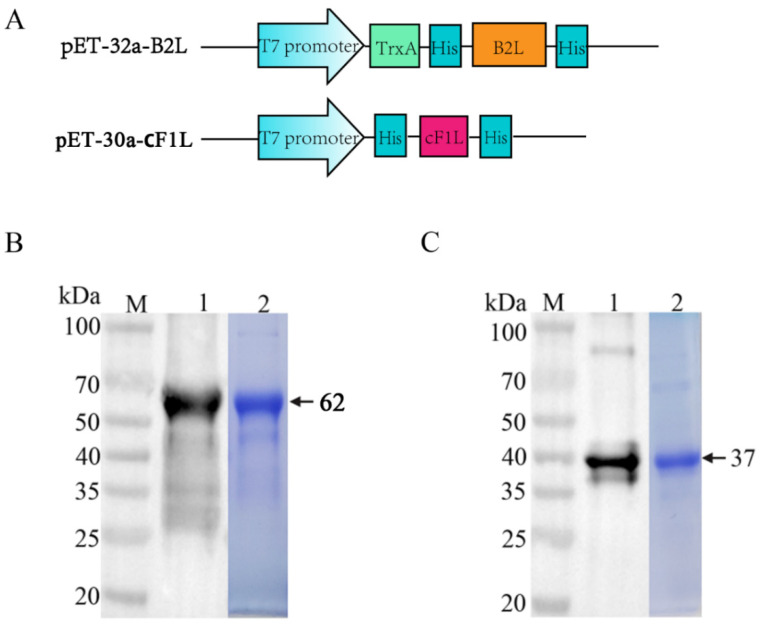
Expression and purification of recombinant *B2L* and c*F1L* protein. (**A**) Schematic diagram of the construction of pET-32a-*B2L* and pET-30a-c*F1L*. (**B**) Western blotting and SDS-PAGE were used to detect the expression and purification of recombinant *B2L* protein. Lane 1: identification of the *B2L* protein expression by Western blotting exploiting mouse anti-B2L protein polyclonal antibodies as primary antibodies; lane 2: the purified recombinant *B2L* protein was detected by SDS-PAGE. (**C**) Western blotting and SDS-PAGE were used to detect the expression and purification of recombinant c*F1L* protein. Lane 1: identification of the c*F1L* protein expression by Western blotting exploiting mouse anti-c*F1L* protein polyclonal antibodies as primary antibodies; lane 2: the purified recombinant c*F1L* protein was detected by SDS-PAGE. M: protein molecular weight marker.

For the expression of ORFV *B2L* and truncated *F1L* protein c*F1L*, pET-32a-*B2L* and pET-30a-c*F1L* were designed and constructed, respectively (Figure 2A). The recombinant *B2L* and c*F1L* proteins were purified in *E. coli* BL21 (DE3), with induction with IPTG; inclusion body protein *B2L* and soluble protein c*F1L* were purified by Ni-affinity column chromatography. Inclusion body protein was inactive, and the *B2L* protein was renatured in a renaturing solution containing 6, 4, 2, and 0 M urea. The refolded recombinant *B2L* protein (62 kDa) and c*F1L* protein (37 kDa) were analyzed using SDS-PAGE (Figure 2B, line 2; Figure 2C, line 2).

Furthermore, polyclonal antibodies of *B2L* and c*F1L* proteins were prepared by immunizing mice four times with purified *B2L* and c*F1L* proteins, and the blood was collected to separate out the serum. The expressions of the above recombinant *B2L* (62 kDa) and c*F1L* protein (37 kDa) were validated by Western blotting (Figure 2B, line 1; Figure 2C, line 1), the results showed that *B2L* and c*F1L* proteins could be specifically reacted with mouse anti-*B2L* and anti-c*F1L* protein polyclonal antibodies, respectively.

### 3.3. Specific Antibody Induction with Different Vaccine Immunization Strategies

**Figure 3:**
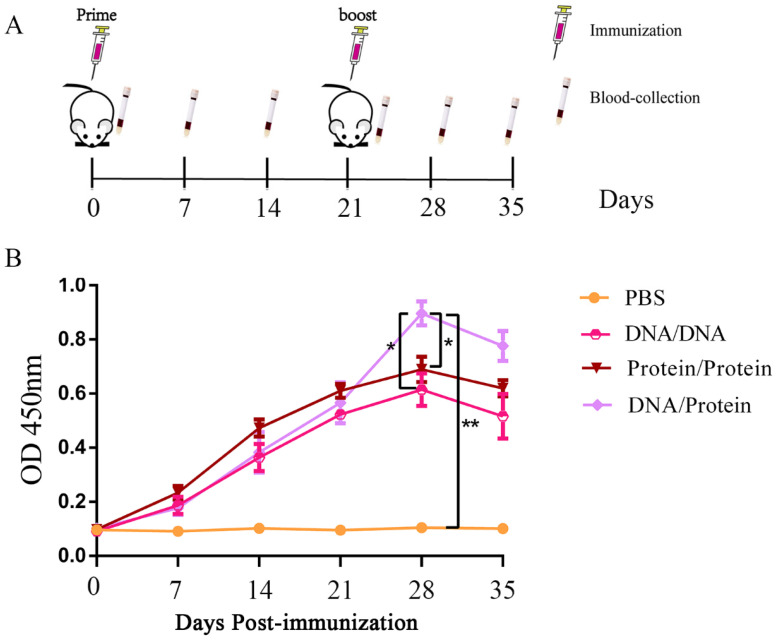
Specific antibody levels in mice immunized with different vaccines. (**A**) Vaccination and blood collection schedule. (**B**) The mouse serum on days 0, 7, 14, 21, 28, and 35 after immunization was collected and inactivated at 56 °C for 30 min. The level of specific IgG in the mice was assessed by indirect ELISA with the purified *B2L* and c*F1L* proteins. Data are expressed as mean ± standard error (*n* = 10). OD_450nm_, optical density at 450 nm, * *p* < 0.05 and ** *p* < 0.01.

In order to study the level of humoral immunity in mice induced by different immunization strategies, the mice were immunized on days 0 and 21. Mouse serum on days 0, 7, 14, 21, 28, and 35 after immunization was collected (Figure 3A), and the levels of specific antibodies in mice were evaluated by indirect ELISA. The results showed that specific antibodies were produced in all vaccine-immunized groups, with a peak level at day 7 after boost immunization. Importantly, on day 28, the level of serum-specific antibody in the DNA prime-protein boost immunization group was significantly higher (*p* < 0.05) than those of the subunit and DNA vaccine-immunized groups; while no effective antibodies were detected in the PBS-inoculated group (Figure 3B). The above data indicate that all immunization strategies induced robust humoral immune responses, and the DNA prime-protein boost vaccination triggered a better immune effect.

**Figure 4:**
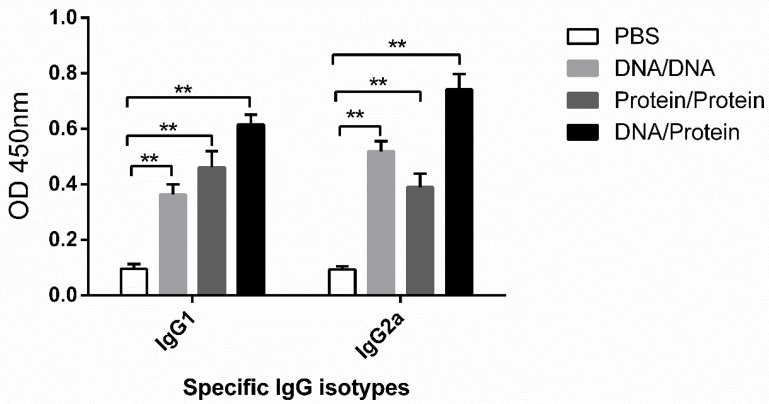
Levels of IgG1 and IgG2a subtypes of IgG antibody in serum of mice after vaccination. The serum of each group of mice was collected 14 days after the booster immunization, and the levels of IgG1 and IgG2a subtypes were evaluated by indirect ELISA. Each group of data is expressed as mean ± standard error (*n* = 10). The absorbance value was measured at OD_450nm_. ** *p* < 0.01.

As shown in Figure 4, the expression levels of IgG antibody IgG1 and IgG2a subtypes in mouse serum were examined on day 14 after boost immunization. The result showed that the levels of IgG1 and IgG2a antibodies in all vaccine-immunized groups were significantly higher (*p* < 0.01) than in the PBS-inoculated group. Additionally, we also found that the ratio of IgG2a/IgG1 in the DNA vaccine-immunized group and the DNA prime-protein boost vaccine-immunized group was higher than 1, indicating that the Th1-type immune response may play a dominant role in this context. Meanwhile, the IgG2a/IgG1 ratio of the subunit vaccine-immunized group was less than 1, which mainly induced a Th2-type immune response (Supplementary Materials Figure S1).

### 3.4. Lymphocyte Proliferation in Different Immune Groups

**Figure 5:**
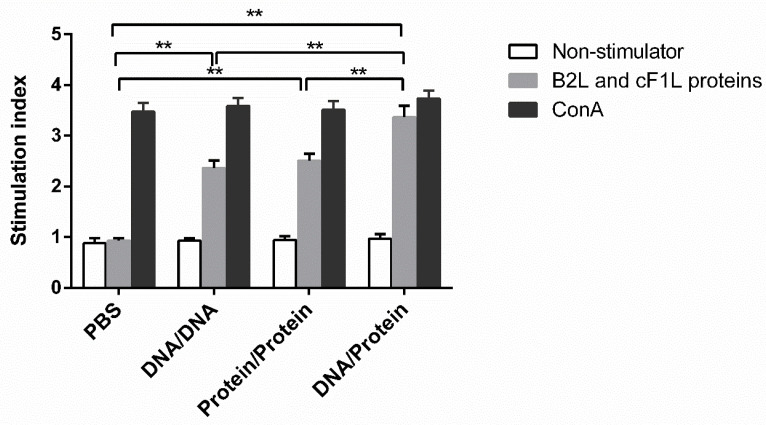
Splenic lymphocyte proliferation reaction of mice in each group two weeks after enhanced immunization. Splenocytes were isolated 14 days after the boost immunization and stimulated with *B2L* and c*F1L* proteins. Lymphocyte proliferation response was measured and expressed as the stimulation index (SI). Stimulation index SI = OD experimental group-OD background /OD negative control group-OD background. Each group of data is expressed as mean ± standard error (*n* = 5), ** *p* < 0.01.

On day 14 after boost-immunization, the proliferative ability of spleen lymphocytes in each vaccine immunization group was measured by MTT colorimetry, as shown in Figure 5. The stimulation index of all vaccine-immunized groups after stimulation with *B2L* and c*F1L* proteins was significantly higher (*p* < 0.01) than in the PBS-inoculated group. Notably, the stimulation index of the DNA prime-protein boost immunization group was significantly higher (*p* < 0.01) than that of the DNA and subunit vaccine-immunized groups. No obvious cell proliferation was detected in the PBS-inoculated group incubated with RPMI-1640 medium. These results indicate that the DNA prime-protein boost immunization group could induce a stronger lymphocyte proliferation in mice.

### 3.5. Cytokine Response after Vaccine Immunization

**Figure 6:**
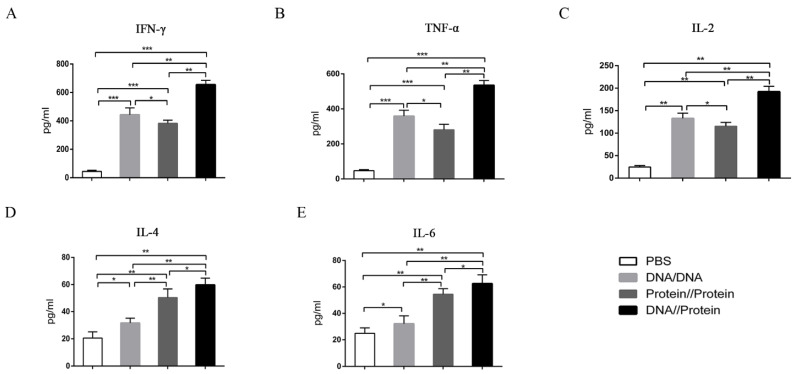
Detection of various cytokines in mouse splenocyte cultures on day 35 after immunization. Splenocytes were isolated 14 days after the last immunization, and the concentrations of IFN-γ (**A**), TNF-α (**B**), IL-2 (**C**), IL-4 (**D**), and IL-6 (**E**) in the culture supernatant were detected using an ELISA kit. Data are expressed as mean ± standard error (*n* = 10). * *p* < 0.05, ** *p* < 0.01 and *** *p* < 0.001.

On day 35 after booster-immunization, to further evaluate the response ability of spleen cells from vaccine-immunized mice, the levels of cytokines production in the supernatant were detected after the stimulation of spleen lymphocytes with purified *B2L* and c*F1L* proteins. As shown in Figure 6A–C, the levels of Th1-type cytokines (IFN-γ, TNF-α, and IL-2) in all vaccine-immunized groups were significantly increased (*p* < 0.01 or *p <* 0.001) when compared with the PBS-inoculated group. Moreover, the levels of these cytokines in the DNA vaccine-immunized group were significantly higher (*p* < 0.05) than that of the subunit vaccine-immunized group. Notably, the levels in the DNA prime-protein boost immunization group were the highest, which were significantly higher (*p* < 0.01) than those in the other two vaccine-immunized groups (Figure 6A–C).

In addition, as shown in Figure 6D,E, the levels of Th2-type cytokines (IL-4 and IL-6) in all immunization groups were significantly higher than (*p* < 0.05 or *p <* 0.01) that in the PBS-inoculated group. The levels of these cytokines in the subunit vaccine-immunized group were significantly higher than (*p* < 0.01) that in the DNA vaccine-immunized group. Interestingly, the cytokine production in the DNA prime-protein boost immunization group was significantly higher than that in the DNA (*p* < 0.01) and subunit vaccine-immunized group (*p* < 0.05) (Figure 6D,E).

## 4. Discussion

In the present study, we constructed a DNA vaccine expressing *B2L*/*F1L* fusion protein, and expressed *B2L* and c*F1L* proteins as a subunit vaccine. Then we performed a heterologous DNA prime-subunit boost strategy to evaluate the immune response. We found that this heterologous prime-subunit boost strategy induced stronger humoral and cellular immune responses. On day 14 after the booster immunization, we further evaluated the safety of the vaccine. All organs were normal (Supplementary Materials Figure S2) and the vaccination was safe.

Developing effective vaccines is an important task for the control of the spread of Orf disease and would protect animals from repeat infection. Several studies have demonstrated that the DNA vaccine prime-subunit vaccine boost strategy is more effective than DNA or subunit vaccines alone in eliciting immune responses. Reports have shown that DNA vaccine priming-subunit vaccine booster immunization achieved good immune effects against MERS-CoV [22] and Coxsackie virus [26], while the levels of specific antibodies for these viruses were significantly higher than that induced by the DNA or subunit vaccines alone.

DNA vaccines with important ORFV immunogenic genes have also been studied, but DNA vaccine alone displayed poor antigenicity [15]. In our study, by exploring the effect of a DNA prime-subunit boost strategy, we were able to overcome the potential disadvantages of DNA vaccine immunization. We showed that the antibody level induced by the DNA prime-subunit boost group was higher than in other vaccination groups, indicating that the heterologous prime-boost approach was more effective in enhancing the humoral immune response. We also showed that the heterologous prime-boost group caused a stronger lymphocyte proliferation and higher cytokine levels, indicating an enhanced cellular immune response. Of note, although WT ORFV was not able to cause infectious disease in mice, there is still a need to evaluate immune stimulating capacity and the safety of the vaccination strategy in mice. From our data, we suggest that this kind of prime-boost strategy is worthy of conducting a small-scale clinical trial of sheep immunity, to evaluate its safety and effectiveness.

## 5. Conclusions

In conclusion, we found that an immunization strategy using DNA vaccine priming and subunit vaccine boosting induced strong humoral and cellular immune responses, and our findings open up new ideas for the future development of Orf vaccines.

## Figures and Tables

**Table 1:** Primer sequences used in the study.

Name	Primer Sequence (5′–3′)
32-011	F1: TCCGTCGAC AAGCTT GCATGTGGCCGTTCTCCTCC R1: GTGGTGGTG CTCGAG ATTTATTGGCTTGCAGAAC
30-059	F2: TCCGTCGAC AAGCTT GCATGGATCCACCCGAAATCA R2: GTGGTGGTG CTCGAG CGTCTTCACCTGTATGTAG
3.1-011-P2A-059	F3: TTTAAACTT AAGCTT GCCACC ATGTGGCCGTTCTCCTCC R3: GACATCTCCGGCTTGTTTCAGCAGAGAGAAGTTTGTTGCATTTATTGGCTTGCAGAACTC F4: CAAGCCGGAGATGTCGAAGAGAATCCTGGACCGATGG R4: ATCCACCCGAAATCACGGCCCCCTCTAGACTCGAGTCACACGATGGCCGTGACCAG
3.1-HA011-P2A-Flag059	F5: TTTAAACTT AAGCTT GCCACC ATGTACCCATACGACGTCCCAGACTACGCT ATGGATCCACCCGAAATC R5: ATCTCCGGCTTGTTTCAGCAGAGAGAAGTTTGTTGCATTTATTGGCTTGCAGAACTCCGA F6: AAACAAGCCGGAGATGTCGAAGAGAATCCTGGACCGATGGATTACAAGGATGACGACGATAAG ATGTGGCCGTTCTCCTCC
C3-mCherry-011	F7: TACTCAGAT CTCGAG ATGTGGCCGTTCTCCTCCATC R7: GCAGAATTCG AAGCTT ATTTATTGGCTTGCAGAAC
mCherry	F8: CCAATAAAT AAGCTT GCAACAAACTTCTCTCTGCTG R8: TCGACTGCA GAATTC CTTGTACAGCTCGTCCATG
C3-mCherry-059	F9: CTGTACAAG GAATTC ATGGATCCACCCGAAATCACG R9: GTGGATCCC GGGCCC TCACACGATGGCCGTGACC

Note: red indicates HA and Flag tag sequence; the underlined mark is the sequence of enzyme digestion site; double underscores are Kozak sequences.

**Table 2:** Inoculation details of mice.

Groups	Prime (0 day)	Boost (21 days)	Immune Dose
PBS	PBS	PBS	100 μL/100 μL
DNA/DNA	pcDNA3.1-*B2L*-P2A-*F1L*	pcDNA3.1-*B2L*-P2A-*F1L*	100 μg/100 μg
Protein/Protein	*B2L*/c*F1L* proteins	*B2L*/c*F1L* proteins	40 μg/40 μg
DNA/Protein	pcDNA3.1-*B2L*-P2A-*F1L*	*B2L*/c*F1L* proteins	100 μg/40 μg

## Data Availability

Data sources of the participants are not publicly available.

## Supplementary Materials

The following supporting information can be downloaded at: https://www.mdpi.com/article/10.3390/vaccines10091499/s1, Figure S1: The ratio of IgG2a/IgG1. In order to more intuitively determine the type of T helper responses, the value of OD IgG2a/OD IgG1 was calculated. Figure S2: Micrographs of HE-stained mouse heart, liver, lung, and kidney after the booster immunization. MI: myocardial interstitium. MC: cardiomyocytes. LC: liver cell. HL: hepatic lobules. Al: alveoli. Br: bronchus. RC: renal corpuscle. Magnification 200×. Bar: 50 μm. Figure S3: Uncropped Western blot. A and B: OFTu cells transfected with pcDNA3.1-HA-*B2L*-P2A-Flag-*F1L* plasmid were quantified and normalized to GAPDH. C: the purified recombinant *B2L* protein, the intden of 62 kDa band is about 122322. D: the purified recombinant c*F1L* protein, the intden of 37 kDa band is about 49511. Figure S4: Corresponding to Figure S1, uncropped Western blots are unlabeled.

## References

[1] Kassa T. (2021). A Review on Human Orf: A Neglected Viral Zoonosis. Res. Rep. Trop. Med..

[2] Li H., Zhu X., Zheng Y., Wang S., Liu Z., Dou Y., Li H., Cai X., Luo X. (2013). Phylogenetic analysis of two Chinese orf virus isolates based on sequences of B2L and VIR genes. Arch. Virol..

[3] Wang R., Wang Y., Liu F., Luo S. (2019). Orf virus: A promising new therapeutic agent. Rev. Med. Virol..

[4] Martins M., Rodrigues F.S., Joshi L.R., Jardim J.C., Flores M.M., Weiblen R., Flores E.F., Diel D.G. (2021). Orf virus ORFV112, ORFV117 and ORFV127 contribute to ORFV IA82 virulence in sheep. Vet. Microbiol..

[5] Chi X., Zeng X., Luo S. (2017). Diagnosis and phylogenetic analysis of a multifocal cutaneous orf virus with mixed bacterial infection outbreak in goats in Fujian province, China. Arch. Virol..

[6] Abrahão J.S., Campos R.K., Trindade G.S., Guedes M.I., Lobato Z.I., Mazur C., Ferreira P.C., Bonjardim C.A., Kroon E.G. (2009). Detection and phylogenetic analysis of Orf virus from sheep in Brazil: A case report. Virol. J..

[7] Nandi S., De U.K., Chowdhury S. (2011). Current status of contagious ecthyma or orf disease in goat and sheep—A global perspective. Small Rumin. Res..

[8] Hosamani M., Scagliarini A., Bhanuprakash V., McInnes C.J., Singh R.K. (2009). Orf: An update on current research and future perspectives. Expert Rev. Anti Infect. Ther..

[9] Peralta A., Robles C.A., Micheluod J.F., Rossanigo C.E., Martinez A., Carosio A., König G.A. (2018). Phylogenetic Analysis of ORF Viruses From Five Contagious Ecthyma Outbreaks in Argentinian Goats. Front. Vet. Sci..

[10] Buchan J. (1996). Characteristics of orf in a farming community in mid-Wales. BMJ.

[11] Al-Qattan M.M. (2011). Orf infection of the hand. J. Hand Surg. Am..

[12] Chan K.W., Yang C.H., Lin J.W., Wang H.C., Lin F.Y., Kuo S.T., Wong M.L., Hsu W.L. (2009). Phylogenetic analysis of parapoxviruses and the C-terminal heterogeneity of viral ATPase proteins. Gene.

[13] Gallina L., Scagliarini A., Ciulli S., Prosperi S. (2004). Cloning and expression of the Orf virus F1L gene: Possible use as a subunit vaccine. Vet. Res. Commun..

[14] Yogisharadhya R., Kumar A., Bhanuprakash V., Shivachandra S.B. (2018). Evaluation of a recombinant major envelope protein (F1L) based indirect- ELISA for sero-diagnosis of orf in sheep and goats. J. Virol. Methods.

[15] Zhao K., He W., Gao W., Lu H., Han T., Li J., Zhang X., Zhang B., Wang G., Su G. (2011). Orf virus DNA vaccines expressing ORFV 011 and ORFV 059 chimeric protein enhances immunogenicity. Virol. J..

[16] Jiang W., Jiang P., Li Y., Tang J., Wang X., Ma S. (2006). Recombinant adenovirus expressing GP5 and M fusion proteins of porcine reproductive and respiratory syndrome virus induce both humoral and cell-mediated immune responses in mice. Vet. Immunol. Immunopathol..

[17] Wang S., Yang G., Nie J., Yang R., Du M., Su J., Wang J., Wang J., Zhu Y. (2020). Recombinant E rns -E2 protein vaccine formulated with MF59 and CPG-ODN promotes T cell immunity against bovine viral diarrhea virus infection. Vaccine.

[18] Saha R., Killian S., Donofrio R.S. (2011). DNA vaccines: A mini review. Recent Pat. DNA Gene Seq..

[19] Pitcovski J., Gruzdev N., Abzach A., Katz C., Ben-Adiva R., Brand-Shwartz M., Yadid I., Ratzon-Ashkenazi E., Emquies K., Israeli H. (2022). Oral subunit SARS-CoV-2 vaccine induces systemic neutralizing IgG, IgA and cellular immune responses and can boost neutralizing antibody responses primed by an injected vaccine. Vaccine.

[20] Khulape S.A., Maity H.K., Pathak D.C., Ramamurthy N., Ramakrishnan S., Chellappa M.M., Dey S. (2019). Evaluation of a fusion gene-based DNA prime-protein boost vaccination strategy against Newcastle disease virus. Trop. Anim. Health Prod..

[21] Li Y., Stirling C.M., Denyer M.S., Hamblin P., Hutchings G., Takamatsu H.H., Barnett P.V. (2008). Dramatic improvement in FMD DNA vaccine efficacy and cross-serotype antibody induction in pigs following a protein boost. Vaccine.

[22] Choi J.A., Goo J., Yang E., Jung D.I., Lee S., Rho S., Jeong Y., Park Y.S., Park H., Moon Y.H. (2020). Cross-Protection against MERS-CoV by Prime-Boost Vaccination Using Viral Spike DNA and Protein. J. Virol..

[23] Petrovsky N., Aguilar J.C. (2004). Vaccine adjuvants: Current state and future trends. Immunol. Cell Biol..

[24] Pollack K.E., Meneveau M.O., Melssen M.M., Lynch K.T., Koeppel A.F., Young S.J., Turner S., Kumar P., Sol-Church K., Mauldin I.S. (2020). Incomplete Freund’s adjuvant reduces arginase and enhances Th1 dominance, TLR signaling and CD40 ligand expression in the vaccine site microenvironment. J. Immunother. Cancer.

[25] Zhang Y., Ma Y., Yang M., Min S., Yao J., Zhu L. (2011). Expression, purification, and refolding of a recombinant human bone morphogenetic protein 2 in vitro. Protein Expr. Purif..

[26] Lan J., Gao Z., Xiong H., Chuai X., Jin Y., Li J., Xian X., Liu G., Xie L., Zhang Y. (2011). Generation of protective immune responses against coxsackievirus B3 challenge by DNA prime-protein boost vaccination. Vaccine.

