# Supplementary material for: Evaluation of the Immune Response Afforded by Combined Immunization with Orf Virus DNA and Subunit Vaccine in Mice
Source: Vaccines (Basel). 2022 Sep 8;10(9):1499. doi: 10.3390/vaccines10091499 (PMC9504141; doi:10.3390/vaccines10091499)
Supplement: Supplementary file 1 [file vaccines-10-01499-s001.zip › vaccines-1855868-supplementary.pdf]

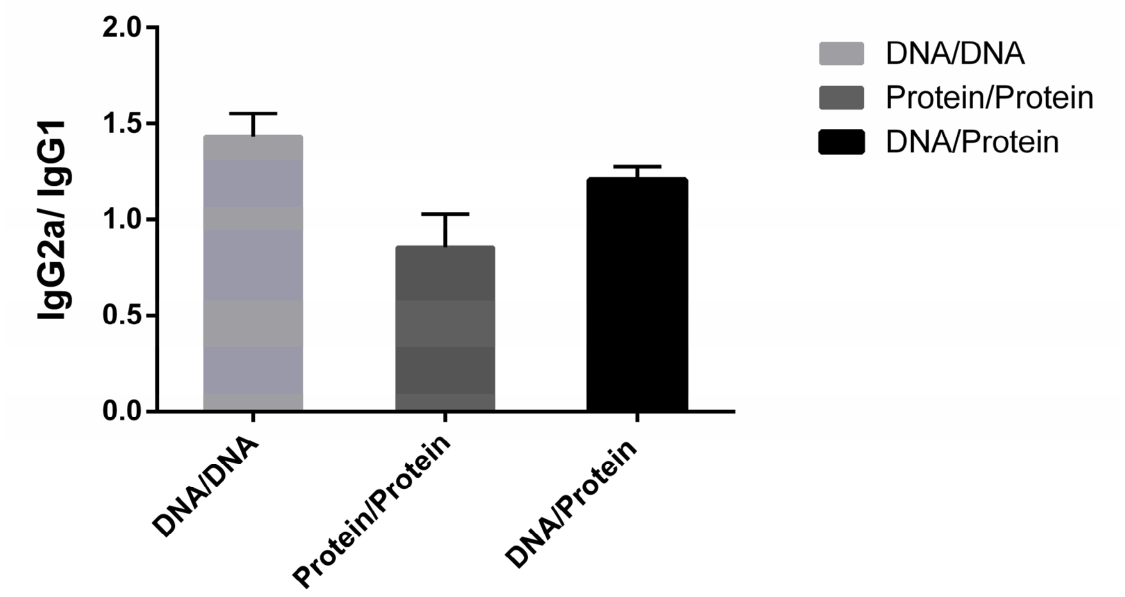

Figure S1. The ratio of IgG2a/IgG1. In order to more intuitively determine the type of T helper responses, the value of OD IgG2a/OD IgG1 was calculated.

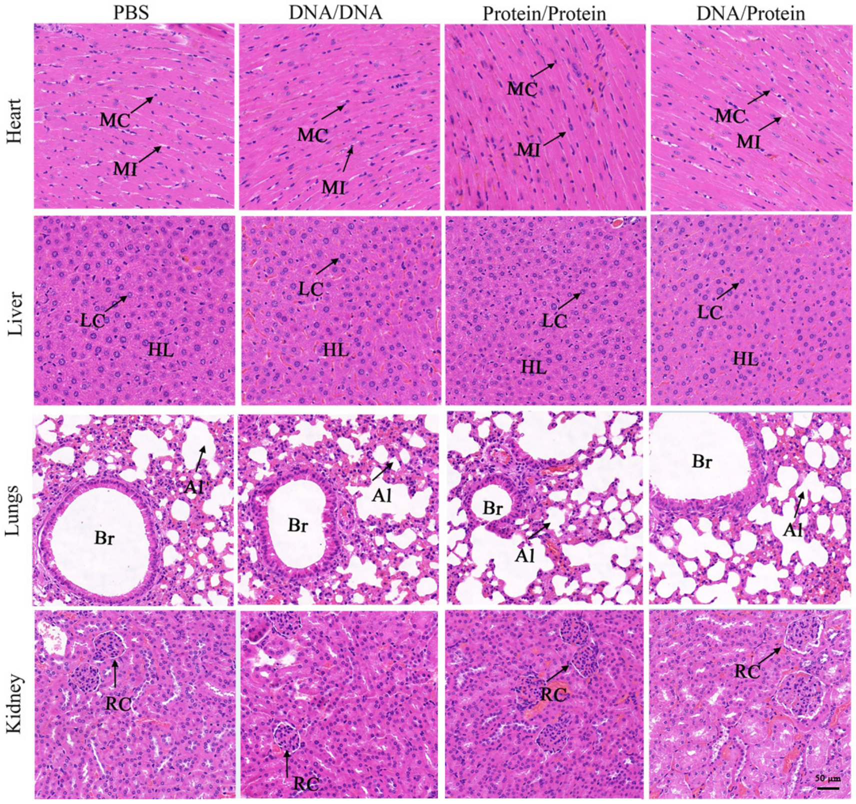

Figure S2. Micrographs of HE-stained mouse heart, liver, lung, and kidney after the booster immunization. MI: myocardial interstitium. MC: cardiomyocytes. LC: liver cell. HL: hepatic lobules. Al: alveoli. Br: bronchus. RC: renal corpuscle. Magnification 200 ×. Bar: 50 μm.

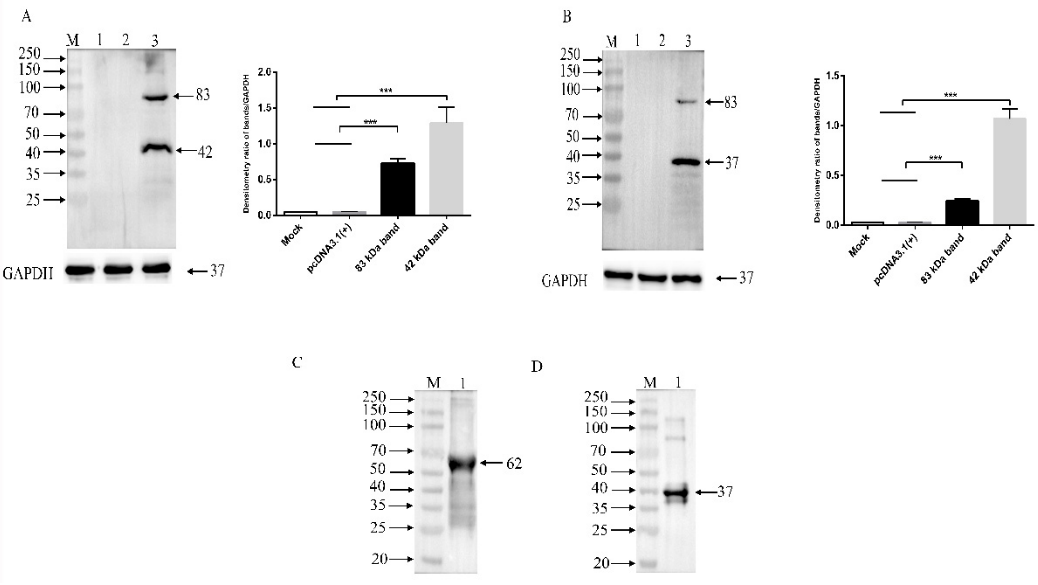

Figure S3. Western blot assay. A and B: OFTu cells transfected with pcDNA3.1-HA-B2L-P2A-Flag-F1L plasmid were quantified and normalized to GAPDH. C: the purified recombinant B2L protein, the intden of 62 kDa band is about 122322. D: the purified recombinant cF1L protein, the intden of 37 kDa band is about 49511.

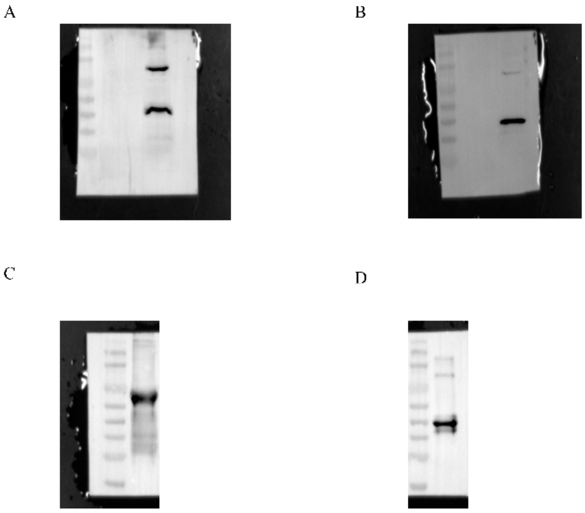

Figure S4. Corresponding to Figure S1, uncropped Western blots
